# Influence of Non-Rubber Components on the Properties of Unvulcanized Natural Rubber from Different Clones

**DOI:** 10.3390/polym14091759

**Published:** 2022-04-26

**Authors:** Nussana Lehman, Akarapong Tuljittraporn, Ladawan Songtipya, Nattapon Uthaipan, Karnda Sengloyluan, Jobish Johns, Yeampon Nakaramontri, Ekwipoo Kalkornsurapranee

**Affiliations:** 1 Division of Physical Science, Faculty of Science, Prince of Songkla University, Hat-Yai 90110, Thailand; nussana1708@gmail.com (N.L.); akarapong04@gmail.com (A.T.); 2 Center of Excellence in Bio-Based Materials and Packaging Innovation, Program of Packaging and Materials Technology, Faculty of Agro-Industry, Prince of Songkla University, Hat-Yai 90110, Thailand; ladawan.so@psu.ac.th; 3 Faculty of Agro-Industry, Prince of Songkla University, Hat-Yai 90110, Thailand; 4 Sino-Thai International Rubber College, Prince of Songkla University, Hat-Yai 90110, Thailand; nuthaipan@gmail.com (N.U.); karnda.seng@gmail.com (K.S.); 5 Department of Physics, Rajarajeswari College of Engineering, Bangalore 560074, India; jobish_johns@rediffmail.com; 6 Sustainable Polymer & Innovative Composite Materials Research Group, Department of Chemistry, Faculty of Science, King Mongkut’s University of Technology Thonburi, Bangkok 10140, Thailand; yeampon.nak@kmutt.ac.th

**Keywords:** fresh natural latex, non-rubber components, clones, cream concentrated latex, hydroxyl ethyl cellulose

## Abstract

Natural rubber from different *Hevea braziliensis* clones, namely RRIM600, RRIT251, PB235 and BPM24, exhibit unique properties. The influences of the various fresh natural rubber latex and cream concentrated latex on the non-rubber components related properties were studied. It was found that the fresh natural rubber latex exhibited differences in their particle size, which was attributed to the non-rubber and unique signature of clones which affect various properties. Meanwhile, the cream concentrated latex showed the protein contents, surface tension, and color of creamed latex to be lower than the fresh natural latex. However, TSC, DRC, viscosity, particle size and green strength of concentrated latex were found to be higher than the fresh natural latex. This is attributed to the incorporation of HEC molecules. Also, the rubber particle size distribution in the RRIM600 clone exhibited a large particle size and uniform distribution, showing good mechanical properties when compared to the other clones. Furthermore, the increased green strength in the RRIM600 clone can be attributed to the crystallization of the chain on straining and chain entanglement. These experimental results may provide benefits for manufacturing rubber products, which can be selected from a suitable clone.

## 1. Introduction

Natural rubber latex (NRL) is obtainable from a rubber plant in the form of latex. Latex is the white milk-like fluid which is obtained by wounding the rubber plant. *Hevea braziliensis* is the most common commercial source of latex today. Fresh natural rubber latex consists of two main components. 25–41% of dry rubber content or hydrocarbon (*cis-*1,4-polyisoprene) and the other non-rubber components consisting of mainly carbohydrates, proteins, lipids, minerals, and salt content in an aqueous serum phase (Table 1) [1,2]. Fresh natural rubber latex can be contaminated by micro-organisms because it contains various nutritious substances otherwise known as non-rubber components [3]. Normally, natural rubber latex spontaneously coagulates shortly after it comes out of the tree. Due to the bacterial attack often occurring on the protein constituents which act as colloidal stabilizers to keep the latex water dispersible, coagulation is prevented [2,4]. In order to facilitate preservation, high amounts of ammonia are added to the latex. Ammonia is added to the latex to maintain in the form of liquid, and this is concentrated either by creaming or centrifuging. The resulting concentration can be transported as a liquid. Fresh natural latex can be transformed to concentrated natural latex in order to maintain the constant quality of concentrated natural latex, and to generate the economic value for latex’s transportation. The concentrated natural latex can be produced by various processes, including evaporation, electrocantation, centrifuging and creaming processes [3].

The creaming process is popular, as it often avoids the use of sophisticated equipment, thus offering a simple and cost-effective route to concentrate the latex. The creaming process is a chemical process involving the addition of creaming agents into the vessels containing field latex to hasten phase separation (upper rubber fraction and lower serum) [5]. The creamed latex generally contains around 50–60% of dry rubber content. It is well-known that concentrated natural rubber latex is used in many applications. Mainly, it has been used in gloves, condoms, toys, balloons, catheters, medical tubing, elastic threads, latex foams, etc. [6,7,8]. NR has outstanding strength, along with excellent dynamic properties, low hysteresis loss, high tensile strength and resistance to forms of fatigue such as chipping, cutting or tearing [9]. However, fresh natural rubber latex from various *Hevea brasiliensis* clones consists of different non-rubber components. Composition of fresh natural latex depends on factors such as soil condition, fertilizer quality, tapping system, season, and, in particular, the natural rubber clonal variety [10]. A previous study on the properties of various *Hevea brasiliensis* clones (i.e., RRIM600, PB235, and RRTI408) clearly showed the difference in protein and lipid contents. The protein and lipid contents, together with gel content, play essential roles in controlling various properties of unvulcanized NR. It is noted that the non-rubber components, especially proteins and phospholipids, have been found to strongly affect the various properties of raw NR and its vulcanizates. For instance, the proteins present in latex play a major role in deciding the properties of latex products such as elasticity, modulus and barrier functions. This relates to the presence of nitrogenous amino acids, which might act as cure accelerators, antioxidants and thermal stabilizers in NR. In addition, the whole lipid content, especially the phospholipids, was found to be inversely proportional to the tack properties of NR and unsaturated fatty acids act as a plasticizer for rubber by lowering the plasticity of NR [11,12]. Furthermore, fresh natural rubber latex from various *Hevea brasiliensis* clones consists of high molecular weight (MW) components and a wide distribution of molar mass (MMD). The molecular structures of two NR clones (i.e., RRIM600 and PB235) were analyzed by size exclusion chromatography (SEC) and it was found that the RRIM600 clone had bimodal MMD, whereas the PB235 clone had unimodal MMD [12]. Therefore, the non-rubber content, especially proteins and lipids, have been found to strongly affect various properties of raw NR and predominantly influence the green strength of un-vulcanized NR. Furthermore, unsaturated fatty acids can act as plasticizers in natural rubber latex [13]. The properties of fresh natural rubber latex are varied (P < 0.01) as a function of clone type, tapping method and climate factors. For example, dry rubber content (DRC) is generally decreased in the beginning of the dry season (May to June), while simultaneously the nitrogen and ash contents (%) are increased in the same period [14].

The present work aimed to study the effect of non-rubber components on the properties of fresh natural latex and creamed concentrated latex. Fresh natural latex from 4 different clones (RRIM600, RRIT251, PB235 and BPM24) were chosen to study their different dry rubber content and molar mass distributions. The yield of fresh natural rubber latex from four different clones is comparatively more upon introducing new genetic varieties of *Hevea Brasiliensis* recommended by the Rubber Research Institute of Thailand (RRIT). The fresh natural latex was collected from plantations in Songkhla province, Thailand, during the late part of tapping season in Thailand (May and June).

The impact of non-rubber components on both biochemical and physicochemical indicators in liquid (latex) and dry states (film) was investigated. Protein contents, molecular weight (MW), DRC, TSC, surface tension, viscosity and morphology were measured for liquid latex, and the mechanical properties of the dry state (unvulcanized natural rubber film) were investigated.

## 2. Materials and Methods

### 2.1. Materials

Natural rubber latex samples from four *Hevea brasiliensis* clones, namely RRIM600, RRIT251, PB235 and BPM24, were collected from plantations in Songkhla province, Thailand, during the late part of tapping season in Thailand (i.e., May–June). 28% of ammonia solution was added as a preservative to the fresh field NR latex and it prevents coagulation of latex. Hydroxyethylcellulose (HEC) was used as a creaming agent purchased from Brenntag Ingredients Public Company Limited (Bangkok, Thailand). Potassium laurate was purchased from Lucky Four Co., Ltd., (Bangkok, Thailand) and used as a pH modifier for the creaming process of fresh field NR latex. 

### 2.2. Preparation of Creamed Concentrated Latex

Creamed concentrated latex was prepared using a formulation, as shown in Table 2. The percentages of dry rubber content of four *Hevea brasiliensis* clones, namely RRIM600, RRIT251, PB235 and BPM24, are found to be 41.0%, 40.8%, 42.5% and 24.0%, respectively. 20% (*w/w*) potassium laurate was added into the treated latex with continuous mechanical stirring for 5 min before incorporation of the creaming agent. The creamed concentrated latex with hydroxyethyl cellulose (HEC) as a creaming agent was dissolved in deionized water at a concentration of 1% (*w/w*). The mixture was thoroughly stirred at 120 rpm for 30 min. The mixture was then incubated at room temperature for 7 days. After 7 days, the latex was found to be separated into two layers, rubber particles at the top and aqueous serum at the bottom. The aqueous serum phase was removed and the upper rubber fraction—the creamed concentrated latex—was finally extracted from the mixture for further investigation.

### 2.3. Characterization and Measurements

#### 2.3.1. Analysis and Testing of the Fresh Natural Latex and Creamed Concentrated Latex

Analysis and testing of latex including the total solid content (TSC) and dry rubber content (DRC) were performed according to ISO 124 and ISO 126, respectively. The viscosity of latex was also measured using a Brookfield digital viscometer, model LVDV - III Ultra, with spindle no. 2 at a speed of 60 rpm. Furthermore, the surface tension was determined by Du Noüy ring method [15]. The measurement was performed by an instrument known as a Tensiometer.

#### 2.3.2. The Protein Contents

The protein content was estimated from its nitrogen content by the Kjeldahl method according to the Association of Official Analytical Chemists (AOAC) and Official Methods of Analysis of Fertilizers (OMAF) [16]. A 0.1 g rubber sample was mixed with a mixture of catalysts (0.65 g) and then digested in concentrated sulfuric acid (H_2_SO_4_) until the rubber was completely digested. Then, an alkaline solution (67% w/v NaOH) was added to the mixture of the digested solution. After that, the solution was distilled, and the distillate was collected in a boric acid solution. Finally, the distillate was titrated with 0.01 N H_2_SO_4_ to determine the ammonia content, allowing for estimating the protein content by multiplying the nitrogen content (mass) with 6.25.

#### 2.3.3. Molecular Weight and Polydispersity Index

Molecular weight (Mw), number-average molecular weight (Mn) and polydispersity index (PDI) of the fresh natural latex were investigated by gel perforation chromatography (GPC) technique (1260 infinity II GPC/SEC MDS, Agilent Technologies, Germany) with a refractive index detector. The solution of fresh natural latex samples with a concentration of 0.001 g/ mL was prepared by using tetrahydrofuran (THF) as a solvent before filtrating through a 0.45 μm membrane. The THF was also applied as a mobile phase with a flow rate of 1.0 mL/min under 40 °C.

#### 2.3.4. Morphological Properties

Particles and particle distribution of the lattices were also analyzed using a laser particle size analyzer, Coulter model LS230 particle size analyzer. Furthermore, morphological properties of the latex particles were examined with a transmission electron microscope (TEM), model Jem 2010, Japan with 160 kV with a magnification of 10,000×. In TEM technique, the latex was first diluted with deionized water to a concentration of 0.025 wt%. An aqueous solution of OsO_4_ (2 wt%) was then added into the diluted latex and allowed to stain the rubber molecule overnight [17]. The stained samples were then examined by TEM.

#### 2.3.5. Mechanical Properties

The tensile testing was performed using a universal testing machine (model H10KS, Hounsfield, UK). The tests were performed with a crosshead speed of 500 mm/min at room temperature using dumbbell-shaped specimens according to ASTM D412. In the case of hardness, the samples were tested using a Shore A durometer (Frank GmbH, Hamburg, Germany) as per ASTM D2240. Furthermore, the color of NRs from various *Hevea brasiliensis* clones was characterized with a Lovibond colorimeter according to ASTM D3157 and with a HunterLab spectrophotometer.

## 3. Results and Discussion

### 3.1. Characterization of Fresh Natural Latex

The physical properties of fresh natural latex from four different *Hevea brasiliensis* clones were measured. Table 3 shows the TSC (total solid content), DRC (dry rubber content), protein contents, surface tension, viscosity and color of different fresh natural latex collected from a variety of clones. TSC, DRC and protein contents showed different results for NRs from various clones. The DRC of RRIM600, RRIT251 and PB235 clones exhibited the same trend (40–42%), whereas the BPM24 clone showed the least value. Moreover, protein contents estimated from Kjeldahl method also showed different values. This is consistent with the observation that clones and the environment affect the metabolism of latex regeneration [18]. The viscosity of the fresh natural latex depends on TSC and DRC due to a high TSC, and DRC may limit the yield by hindering latex flow. In addition, the surface tension is the important parameter that decides the physical properties of fresh natural latex with respect to its intrinsic properties and its applications (wetting and dipping). The results of surface tension of all clones showed the same trend (42–45 mN/m). 

**Figure 1:**
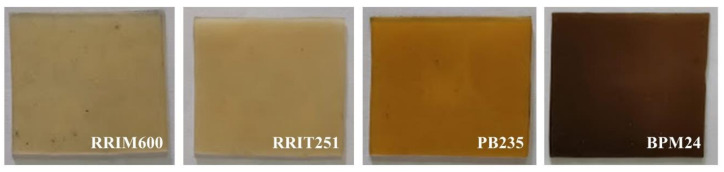
The color of the fresh natural latex from four different *Hevea brasiliensis* clones.

The color of all clones selected were determined with a Lovibond colorimeter and HunterLab spectrophotometer, and they are summarized in Table 3. A photo of the rubber sample is also shown in Figure 1. The yellow color of NR is not only due to the presence of non-rubber constituents but also depends on clonal and seasonal variations, soil types and tapping frequency. The distinctive yellow color in NR has been attributed to the presence of carotenoids. Mostly, the non-rubber components affect the color of products made from it. Therefore, the color change of the samples is likely to arise from the oxidation of non-rubbers such as proteins and lipids [19].

### 3.2. Rubber Particle Size Distribution of the Fresh Natural Latex

**Figure 2:**
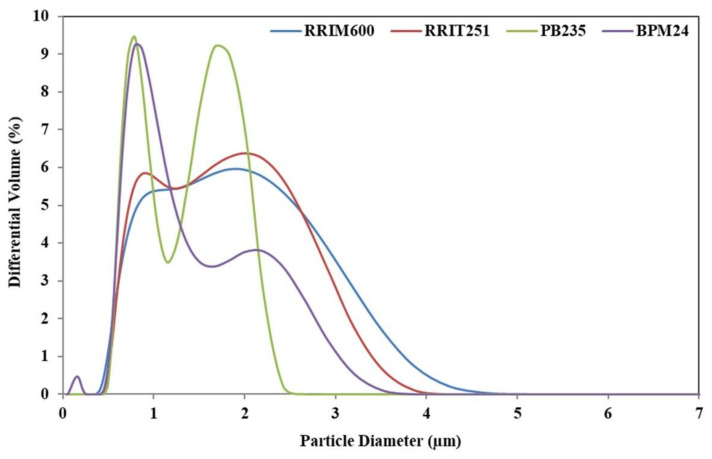
Particle size distribution of the fresh natural latex from four different *Hevea brasiliensis* clones.

Figure 2 shows the particle size distribution of fresh natural latex from four different *Hevea brasiliensis* clones. Results showed that the diameters of rubber particles in all clones varied between 0.40 µm and 5.00 µm. The typical quasi-unimodal particle size distribution for RRIM600 and RRIT251 clones was observed, whereas PB235 and BPM24 clones were bimodal. This confirms the presence of two populations of chains in rubber, leading to determination of the average molar masses, as well as the size and shape of natural rubber molecules. The main difference between the four clones therefore lies in the relative quantity of short chain. It is anticipated that the high number of short chains in the bimodal distribution for PB235 and BPM24 clone yielded many chain ends or terminals of NR molecules and chain ends together with more non-rubber components than the other clones.

**Figure 3:**
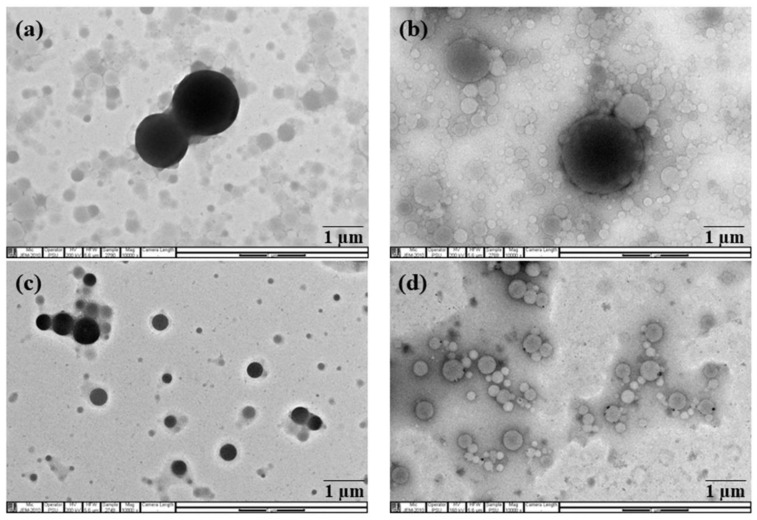
Transmission electron micrographs of (**a**) RRIM600; (**b**) RRIT251; (**c**) PB235; and (**d**) BPM24. (×10,000).

Rubber particle size in RRIM600 and RRIT251 clones are found to be larger in the range between 0.50 µm and 4.50 µm. The fine particles with size lower than 0.50 µm are observed in BPM24 clone together with more portions of larger rubber particles in the range between 0.40 µm and 3.50 µm and shows wider particle size distribution than other clones, whereas PB235 clone shows narrower distribution in the range of 0.45–2.52 µm. The difference in the range of particle size might be attributed to the non-rubbers and the unique signature of the clones [20]. These results are in good agreement with the results of transmission electron micrographs as shown in Figure 3. It is clearly observed that the latex of RRIM600 and RRIT251 clones consist of large particles (Figure 3a,b), while the latex from PB235 and BPM24 clones (Figure 3c,d) consist of both large and small particles. Rubber particles are observed as spherically shaped and some were pear-shaped; regardless, their particle size, either they were large or small rubber particles for all clones selected [21]. It was observed that some elongated particles appeared as a result of aggregation of non-rubber in the analyzed sections of BPM24, consisting of many nodules of non-rubber on the particle surfaces.

### 3.3. Molar Mass Distribution of the Fresh Natural Latex

The molar mass and molar mass distributions (MMD) in the natural rubber play an important role in their bulk properties. The molar mass distribution of natural rubber from RRIM600, RRIT251, PB235 and BPM24 clones are shown in Table 4. MMD, in the case of all clones, confirmed that the rubber from RRIM600 and RRIT251 are bimodal MMD, but the rubber from PB235 and BPM24 clones are unimodal MMD. The properties of NRs from the four clones depend on the relative quantity of short chains. It is anticipated that the high number of short chains in this bimodal distribution yielded many chain ends or terminals of NR molecules [10]. The polydispersity index (PDI) of the BPM24 clone exhibited a superior value when compared to the other clones and exhibited an MWD curve with unimodal distribution with almost constant value skewed in case of low molecular weight. However, the size of rubber particles might influence the molecular weight of their rubber particles in the latex.

### 3.4. Mechanical Properties of Fresh Natural Latex

**Figure 4:**
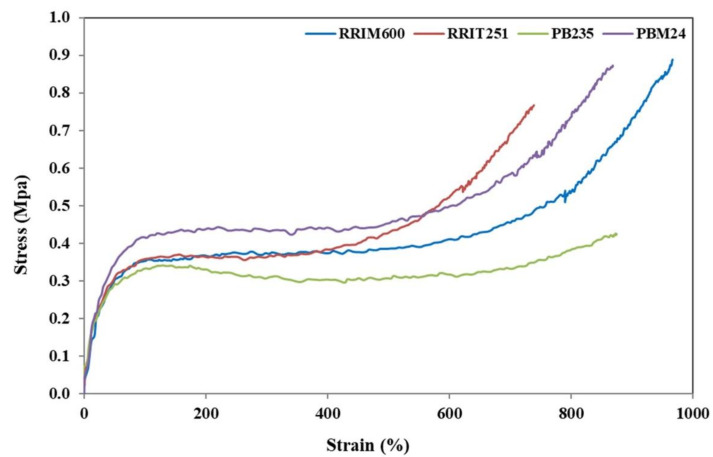
Stress-strain curves of NRs from the four *Hevea brasiliensis* clones.

**Figure 5:**
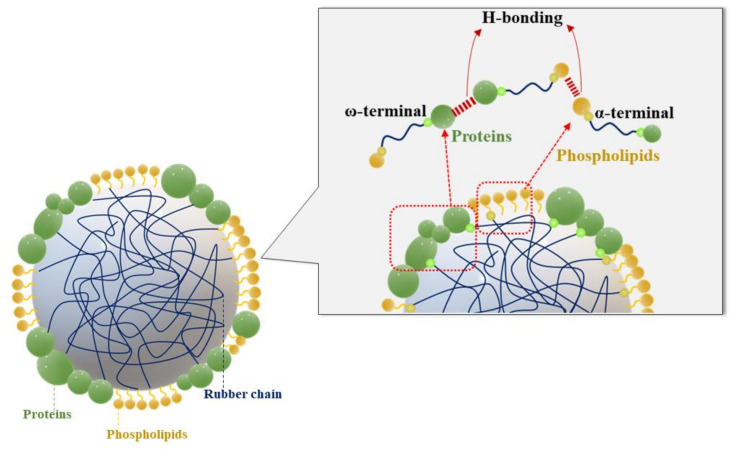
Schematic representation of α- and ω-terminal groups on the rubber latex particle surface.

Table 5 shows the mechanical properties of NR films collected from four different *Hevea brasiliensis* clones. The green strength of elastomers has been commonly attributed to long-chain branching, interactions between polar groups, the presence of a gel, chain entanglements, and crystallization on stretching [22]. The variations in these factors are responsible for the different green strength in the case of NRs from various rubber clones. It was seen that RRIM600, RRIT251 and BPM24 clones showed higher stress at break compared to PB235. This might be attributed to the lower protein content (Table 3) that corresponds to the levels of short chains *cis-1,4*-isoprene. It was presumed that the non-rubber components in rubber molecules caused the formation of loosely crosslinked structures or a gel in natural rubber. Generally, the area under stress-strain curve indicates the toughness of a material. In Figure 4, it is seen that the PB235 clone showed the least area due to the least toughness among the other clones, and this was related to the results reported previously [10,11]. This finding clearly indicates that the non-rubber components (i.e., protein content) acted as the reinforcing filler and enhanced the rigidity of rubber. They also form macro-gel in rubber molecules by providing stronger rubber networks with high moduli, stiffness, and hardness [11,23,24,25]. This indicates that the removal of proteins or non-rubber components lead to reduce the moduli, stress at break and hence the stiffness of rubber [25]. In the previous work, [10,22,26] rubber molecular chains comprise of long-chain isoprene units and the chain ends consist of one protein end group and another phospholipid end group. It can be seen that the α-terminal group with mono- or di-phosphate groups associated with phospholipids, whereas the ω-terminal is a dimethylallyl group that interacts with proteins [26]. In Figure 5, it is clearly seen that these molecules could interact with both the functional terminal groups via hydrogen bonding and ionic bonds derived from metal ions. The proteins in natural rubber are considered to originate branch points by hydrogen bonding, as well as the phospholipids are linked to another phospholipid molecule in other chain ends via hydrogen bonding or ionic bonds derived from metal ions. Moreover, proteins at ω-terminal could interact with other protein molecules through hydrogen bonding [10,26]. In addition, the increase in green strength can be attributed to the number of branch points per chain and chain entanglement. The long chain branching in rubber molecule plays an important role in the higher green strength [22]. It was found that a high polydispersity index in case of RRIM600 and BPM24 clones exhibited the higher green strength. These findings might have resulted from the distribution of larger rubber particles and not that of smaller particles. Thus, it is reasonable to assume that the long chain branching in natural rubber affects the green strength. Therefore, it is concluded that the green strength is related to the non-rubber components, branch structure, chain entanglements, and particle size in the natural rubber molecules. 

### 3.5. Characterization of Cream Concentrated Latex from Four Different Hevea Brasiliensis Clones

**Figure 6:**
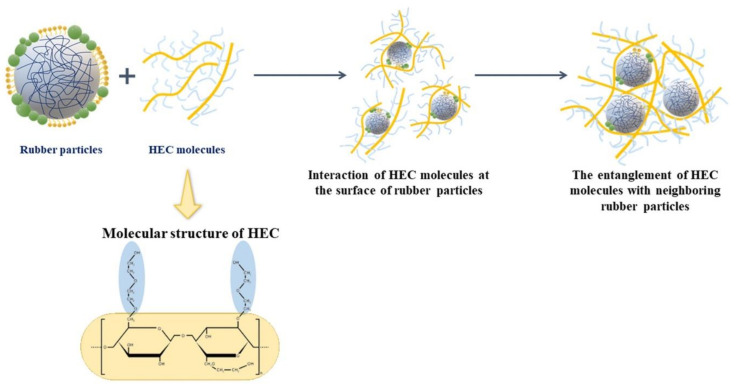
Proposed model for the structures of creamed latex with the incorporation of HEC molecules at the surface of rubber particles.

**Figure 7:**
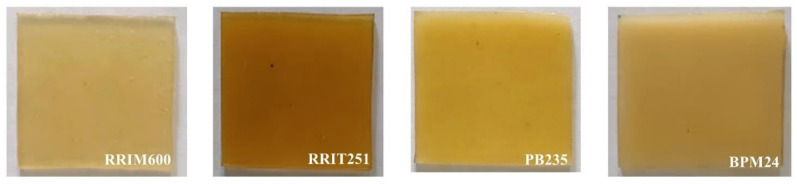
The color of cream concentrated latex from four different *Hevea brasiliensis* clones.

Creaming method is a physicochemical process in which the creaming agent is mixed with fresh natural latex and then a phase separation process (upper rubber fraction and lower serum) is applied [4]. This work is aimed at preparing concentrated latex using hydroxyethyl cellulose (HEC) as a creaming agent. Creamed concentrated latex generally contains the creaming agent, as well as ammonia for preservation. It was found that TSC, DRC, viscosity, and particle size values of the cream concentrated latex are clearly higher than those of fresh natural latex, whereas the protein content, surface tension, and the color exhibited lower values. Table 6 shows the TSC and DRC of creamed concentrated latex, which are increased in the case of all clones when compared to the fresh natural latex. This indicates that hydroxyethyl cellulose (HEC) increases the efficiency to separate rubber particles from the serum solution [2,4]. The incorporation of high-molecular weight hydroxyethyl cellulose (HEC) with higher degree of polarity is responsible for the creaming phenomenon. Thus, hydroxyethyl cellulose (HEC) as a hydrophilic colloid dispersed in aqueous medium and covered the surface of rubber particles. Also, the branched segments of the HEC molecules could be well entangled with the neighboring rubber particles (Figure 6), restricting the movement of the rubber particles and causing a larger rubber particle size that led to an increase in viscosity of the latex [2,27]. It is clear that the creamed concentrated latex from all the clones selected exhibit higher TSC, DRC, particle size and viscosity. Furthermore, during the creaming process, ammonia and potassium laurate soap need to be added in order to prevent coagulation and reduce the viscosity in the latex. It is known that potassium laurate soap is a nonionic surfactant used as stabilizing additives for natural rubber latex. The addition of surfactants during storage modifies the surface of the rubber particles or interface between rubber particles and water by reducing the energy difference between rubber hydrocarbon chains and water. It forms a stable colloidal mixture by reducing the surface tension of all clones selected [28]. Moreover, the addition of creaming agent, ammonia, and surfactant leads to chemical transformations of latex particles along the storage time. Therefore, the amount of non-rubber components or protein should be reduced. The protein content of fresh natural latex is decreased after the creaming process, especially in the case of RRIM600 and PB235 clones reduced the values by 27% and 38%, respectively. Proteins associated with rubber particles are reduced by strong alkali hydrolysis known as saponification reaction [26,27,29,30]. Finally, the color of rubber according to Lovibond colorimeter in creamed concentrated latex collected from all clones showed lighter color compared to the fresh natural latex, especially the BPM24 clone shown in Figure 7. The removal of some non-rubber constituents from the latex during storage may be the reason for this light color. It was found that the RRIM600 clone exhibits more transparency than other clones. Therefore, it was concluded that the properties of cream concentrated latex depend on creaming agent, stabilizing agent, and non-rubber components.

### 3.6. Rubber Particle Size Distribution of Fresh Natural Latex and Cream Concentrated Latex from the RRIM600 Clone

**Figure 8:**
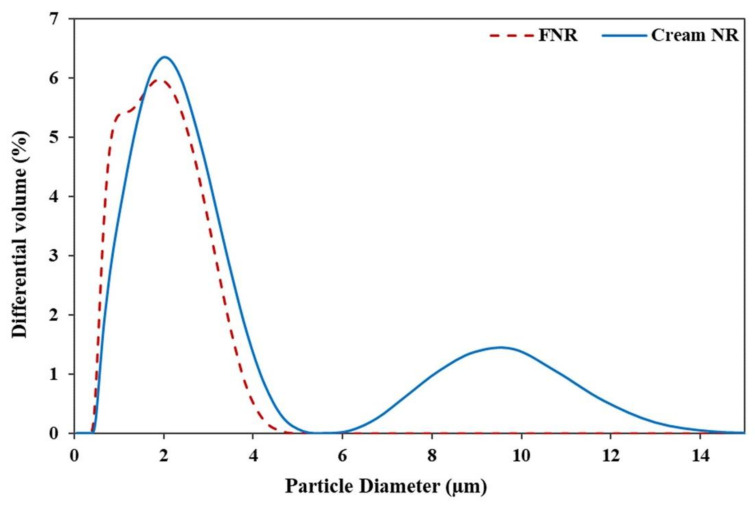
Particle size distribution of the fresh natural latex and cream concentrated latex from the RRIM600 clone.

**Figure 9:**
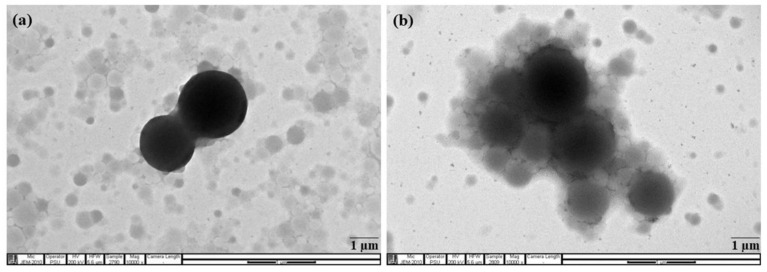
Transmission electron micrographs of (**a**) Fresh natural latex; and (**b**) Cream concentrated latex from the RRIM600 clones. (×10,000).

Figure 8 shows the particle size distribution of fresh natural latex and cream concentrated latex from the RRIM600 clone. The result shows that the diameter of rubber particles in cream concentrated latex are distributed widely in the range of 0.4–15 µm, whereas the fresh natural latex showed a narrower distribution in the range 0.4–5.0 µm. It is clear that the hydroxyethyl cellulose (HEC) molecules covered the surface of rubber particles and might be attributed to all types of rubber particles that can possibly diffuse from the serum into the creamed layers during the creaming process [27]. Thus, the larger size is observed in the rubber particle in the case of concentrated latex. The results are in good agreement with the TEM measurement, as shown in Figure 9. The cream concentrated latex (Figure 9b) showed a greater number of large particles due to the enclosement of HEC and surfactant layer on the surface of particles.

### 3.7. Comparison between the Mechanical Properties of Fresh Natural Latex and Cream Concentrated Latex fromRRIM600 Clone

**Figure 10:**
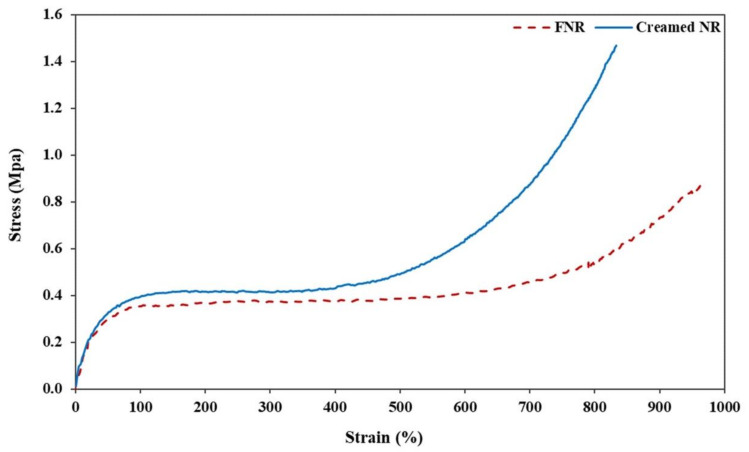
Stress-strain curve of fresh natural latex and cream concentrated latex from RRIM600 clone.

Table 7 shows the green strength, moduli at 100, 300, and 500% elongations, elongation at break, and hardness. Also, Figure 10 shows the stress-strain curve of fresh natural latex, and is compared with the cream concentrated latex. The overall properties of cream concentrated latex were shown to be higher than that of the fresh natural latex, except the elongation at break. This can be explained by the long chain branching in HEC molecules and physical entanglement between HEC and rubber chains. This might be attributed to the crystallization on stretching being related to the number of branch points per chain and chain entanglement [26,31]. This physical entanglement could resist the movement of rubber chains. This result led to the increase in modulus, green strength, and hardness by reducing the elongation at break. Furthermore, the toughness observed from the area under stress-strain curve of the fresh latex exhibit slightly higher than creamed latex. This might be related to the non-rubber components due to the higher non-rubber constituents (proteins and lipids) forming strong structures of loose crosslinks in rubber molecules [10]. Therefore, it is concluded that the green strength is related to the incorporation of high-molecular weight HEC molecules as a creaming agent and non-rubber components, along with rubber molecules.

## 4. Conclusions

Natural rubber was collected from four different *Hevea brasiliensis* clones (i.e., RRIM600, RRIT251, PB235, and BPM24) and the studies showed a variation in their non-rubber components. The amount of non-rubber components in the latex led to better mechanical and physicochemical properties, such as TSC, DRC, viscosity, surface tension, color, particles size, and molar mass distribution. It was found that the RRIM600, RRIT251, and BPM24 clones showed a protein content of about 3.30 wt%, while the PB235 clone exhibited the least protein content. In addition, the rubber particle size distribution in the RRIM600 clone exhibited a large particle size and a uniform distribution. This resulted in better mechanical properties when compared to the other clones.

Furthermore, the cream concentrated latex was successfully prepared using HEC as a creaming agent and potassium laurate as a creaming aid. It was found that the cream concentrated latex showed higher TSC, DRC, viscosity, particle size, and mechanical properties compared to the fresh natural latex. However, except for the surface tension, protein contents and color were found to be lower than the cream concentrated latex. This is attributed to the incorporation of high-molecular weight HEC molecules and surfactant in the cream concentrated latex. Knowledge acquired from this investigation may lead to various applications according to the unique signature of natural rubber from each clone.

## Figures and Tables

**Table 1:** Composition of fresh natural rubber latex [2].

Constituents	% Composition
Rubber particles (cis-1,4-polyisoprene)	30–40
Protein	2.0–3.0
Lipids	0.1–0.5
Resins	1.5–3.5
Ash	0.5–1.0
Sugars	1.0–2.0
Water	55–65

**Table 2:** Formulation is used to prepare creamed concentrated latex.

Chemicals	Quantity, phr
Fresh filed NR latex	100
HEC (1% *w/w* in water)	0.4
20% *w/w* potassium laurate	0.3

**Table 3:** TSC, DRC, protein content, surface tension, viscosity, lovibond comparator and particle size of the fresh natural latex from four different *Hevea brasiliensis* clones.

Properties	*Hevea Brasiliensis* Clones			
	RRIM600	RRIT251	PB235	BPM24
Total solid content (%)	45.30 ± 0.16	43.47 ± 0.13	45.16 ± 0.05	28.03 ± 0.03
Dry rubber content (%)	40.90 ± 3.45	40.82 ± 0.28	42.47 ± 0.58	23.61 ± 1.90
Protein content (wt%)	3.03 ± 0.009	3.33 ± 0.007	2.64 ± 0.004	3.31 ± 0.002
Viscosity (cps) (Spin no.1/Speed 60 rpm)	12.50	11.50	12.00	5.00
Surface tension (mN/m)	45.50 ± 0.70	45.00 ± 1.41	42.00 ± 0.00	45.00 ± 1.41
Lovibond comparator	2.50–3.00	3.00–4.00	8.00–10.00	12.00–14.00
Particle size (µm) (mean)	1.66	1.59	1.27	1.24

**Table 4:** Molecular weight and polydispersity index of fresh natural latex from four different *Hevea brasiliensis* clones.

Hevea Brasiliensis Clones	M_n_ × 10^5^ (g/mol)	M_w_ × 10^6^ (g/mol)	Polydispersity Index (PDI)
RRIM600	3.81	1.96	5.14
RRIT251	3.71	1.68	4.53
PB235	4.34	2.02	4.66
BPM24	1.37	1.09	7.92

**Table 5:** Mechanical properties of NR from fresh natural latex collected from four different *Hevea brasiliensis* clones.

Properties	*Hevea Brasiliensis* Clones			
**RRIM600**	**RRIT251**	**PB235**	**BPM24**
100% modulus (MPa)	0.36 ± 0.01	0.37 ± 0.04	0.32 ± 0.01	0.40 ± 0.02
300% modulus (MPa)	0.38 ± 0.01	0.39 ± 0.06	0.34 ± 0.04	0.42 ± 0.02
500% modulus (MPa)	0.40 ± 0.02	0.42 ± 0.08	0.36 ± 0.01	0.45 ± 0.04
Green strength (MPa)	0.86 ± 0.07	0.73 ± 0.09	0.47 ± 0.07	0.89 ± 0.06
Elongation at break (%)	969 ± 13	884 ± 4	850 ± 45	877 ± 30
Hardness (Shore A)	15.0 ± 0.8	15.5 ± 0.5	13.0 ± 0.8	14.0 ± 0.5

**Table 6:** TSC, DRC, protein contents, surface tension, viscosity, Lovibond comparator and particles size of the cream concentrated latex from four different *Hevea brasiliensis* clones.

Properties	*Hevea Brasiliensis* Clones			
	RRIM600	RRIT251	PB235	BPM24
Total solid content (%)	56.75 ± 0.02	54.46 ± 0.05	66.30 ± 0.05	49.93 ± 0.15
Dry rubber content (%)	54.91 ± 0.06	51.53 ± 1.27	64.80 ± 0.02	48.01 ± 1.81
Protein content (wt%)	2.20 ± 0.002	2.93 ± 0.009	1.64 ± 0.004	2.60 ± 0.001
Viscosity (cps)(Spin no.1/Speed 60 rpm)	46.00	41.00	75.50	7.00
Surface tension (mN/m)	41.50 ± 0.71	41.00 ± 0.00	39.00 ± 0.00	38.50 ± 0.70
Lovibond comparator	2.00–2.50	3.50–5.00	7.00–8.00	5.00–7.00
Particle size (µm) (mean)	1.90 ± 0.00	1.79 ± 0.70	1.52 ± 0.70	1.22 ± 0.64

**Table 7:** Mechanical properties of fresh natural latex and cream concentrated latex from RRIM600 clone.

Properties	Fresh Natural Latex	Cream Concentrated Latex
100% modulus (MPa)	0.36 ± 0.010	0.39 ± 0.005
300% modulus (MPa)	0.38 ± 0.010	0.42 ± 0.005
500% modulus (MPa)	0.40 ± 0.015	0.51 ± 0.012
Green strength (MPa)	0.86 ± 0.070	1.47 ± 0.030
Elongation at break (%)	969 ± 13	834 ± 4
Hardness (Shore A)	13.0 ± 0.8	27.0 ± 0.5

## Data Availability

Not applicable.
